# Predicting Return-to-Manual Performance in Lower- and Higher-Degree Automation

**DOI:** 10.1177/00187208251323101

**Published:** 2025-02-27

**Authors:** Natalie Griffiths, Vanessa K. Bowden, Serena Wee, Shayne Loft

**Affiliations:** 1 2720 The University of Western Australia, Australia

**Keywords:** automation, air traffic control, trust in automation, workload, human–automation teaming

## Abstract

**Objective:**

To examine operator state variables (workload, fatigue, trust in automation, task engagement) that potentially predict return-to-manual (RTM) performance after automation fails to complete a task action.

**Background:**

Limited research has examined the extent to which within-person variability in operator states predicts RTM performance, a prerequisite to adapting work systems based on expected performance degradation/operator strain. We examine whether operator states differentially predict RTM performance as a function of degree of automation (DOA).

**Method:**

Participants completed a simulated air traffic control task. Conflict detection was assisted by either a higher- or lower-DOA. When automation failed to resolve a conflict, participants needed to prevent that conflict (i.e., RTM). Participants’ self-reported workload, fatigue, trust in automation, and task engagement were periodically measured.

**Results:**

Participants using lower DOA were faster to resolve conflicts (RTM RT) missed by automation than those using higher DOA. DOA did not moderate the relationship between operator states and RTM performance. Collapsed across DOA, increased workload (relative to participants’ own average) and increased fatigue (relative to sample average, or relative to own average) led to the resolution of fewer conflicts missed by automation (poorer RTM accuracy). Participants with higher trust (relative to own average) had higher RTM accuracy.

**Conclusions:**

Variation in operator state measures of workload, fatigue, and trust can predict RTM performance. However, given some identified inconsistency in which states are predictive across studies, further research is needed.

**Applications:**

Adaptive work systems could be designed to respond to vulnerable operator states to minimise RTM performance decrements.

## Introduction

Automation expands work system capability but can fail for a variety of reasons (see Skraaning & Jamieson, 2024, for a taxonomy of automation failure). Thus, human operators are required to supervise automation to intervene (i.e. return-to-manual; RTM) if it fails to perform as intended. Unfortunately, operators can be slower and/or less accurate to RTM control after automation failure compared to when performing the task without automated support. This is theorised to result from impaired situation awareness (SA; Endsley & Kiris, 1995) and is more likely to occur when a higher ‘degree of automation’ (DOA) fails (Onnasch et al., 2014). The concept of DOA (Wickens et al., 2010) describes the level of responsibility of automation (Sheridan et al., 1978) across four stages of information processing: information acquisition, information analysis, decision recommendation, and action execution (Parasuraman et al., 2000). The combination of higher levels and higher processing stages constitutes higher DOA. As expanded below, a meta-analysis by Onnasch et al. reported that as DOA increased^1^, workload decreased and routine performance improved, but SA and RTM performance degraded (termed the Lumberjack effect; ‘the higher the trees, the harder they fall’).

Workload varies as a function of task demands and operator capacity to meet those demands (Hart & Wickens, 1990). Elevated workload can be associated with operator fatigue (Saxby et al., 2013) and experienced as a cumulative disinclination toward task effort (Grandjean, 1979). Both high workload and high fatigue can degrade performance (Hancock & Matthews, 2019; Loft et al., 2023), the former is in line with the Onnasch et al. (2014) meta-analysis and subsequent research indicating that higher DOA can reduce workload and improve performance (Tatasciore et al., 2020, 2022).

However, higher DOA can degrade SA and RTM performance (Onnasch et al., 2014; though exceptions exist, see Endsley & Kaber, 1999; Jamieson & Skraaning, 2020; Tatasciore et al., 2020). Higher DOAs may reduce operator task engagement if low task demands cause underload (Young & Stanton, 2002a, 2002b), decreasing the extent to which operators process information related to automated task(s) (Parasuraman & Manzey, 2010; Wickens et al., 2015), impairing SA/RTM performance.

While lower DOA does not reduce workload and improve performance as much as higher DOA, it can reduce costs. As lower DOA requires more manual input, operators are typically less complacent (McGuirl & Sarter, 2006) and maintain better SA (Endsley & Kiris, 1995; Manzey et al., 2012). Subsequently, lower DOA may be preferable in operational contexts where it is important for operators to RTM effectively. However, lower DOA can sacrifice system capacity and cause overload and fatigue.

One protective factor for RTM decrements is to have *calibrated trust* in automation (Lee & See, 2004). Trust is impacted by a vast range of operator/contextual factors, but perceived automation reliability is influential (Hoff & Bashir, 2015). The Carter et al. (2024) Human-Automation Trust Expectation Model proposed that trust is dynamically calibrated (i.e. modified over time; de Visser et al., 2020; Yang et al., 2023) as operators learn to predict automation reliability. Increased trust predicts reliance on automation (Hussein et al., 2020; Rovira et al., 2007), but performance outcomes of increased reliance depend on the degree to which increased trust is calibrated (Carter et al., 2024; Lee & See, 2004).

A concept for balancing the relative benefits/costs of varying DOA is that of adaptive (work) systems (Feigh et al., 2012; Kaber & Riley, 1999) that can adapt function allocation (e.g. decreasing DOA) or make other adaptations (modifying task scheduling and information presentation) to meet the changing needs of operators (e.g. altered task load and task priorities; for a classification of adaptive system triggers, see Feigh et al., 2012). In the current study we focus on *operator-based triggers*, examples of which are variation in operator performance (Calhoun et al., 2011) or physiology (Wilson & Russell, 2007). Performance triggers are reactive. Physiological triggers are proactive but are not necessarily reliable/valid indicators of cognitive state (Charles & Nixon, 2019).

Another potential trigger for adapting DOA is self-reported variation in operator state – the cognitive, energetic processes that constrain performance (Nickel et al., 2006). Operators can expend compensatory effort to maintain performance, but this can be cognitively taxing (Hockey, 1997) and reduce capacity to respond to future task demands (Loft et al., 2007; Nickel et al., 2006). Detecting vulnerable operator cognitive states may predict performance degradation and allow work system adaptations. For instance, switching to using higher DOAs could help operators cope with increased task demands, while switching to lower DOAs may help operators at risk of performance deficits (i.e. adaptive function allocation), or other adaptations could be triggered (e.g. task scheduling).

### Using Cognitive States to Predict Return-to Manual Performance

RTM performance deficits can manifest in the failure to manually complete a task not completed by automation (*RTM accuracy*), and/or a greater time taken for participants to manually complete a task not completed by automation (*RTM response time* [*RT*]).

Preliminary evidence suggests that some aspects of operator state can predict RTM performance. Griffiths et al. (2022) found that variations in self-reported operator fatigue and trust in automation, at the between- and/or within-person levels of analysis, predicted RTM RT when higher DOA failed in a simulated air traffic control (ATC) task. At the *between-person level*, an operator’s state reflects how they are perceiving/responding to task demands relative to others (e.g. individuals experiencing higher-than-average workload performing more poorly than those experiencing lower-than-average workload). At the within-person level, changes in operator state reflect how an individual is perceiving/responding to task demands relative to themselves (e.g. an individual’s subsequent RTM performance may degrade following a prior increased trust in automation relative to their average trust).

Within-person variation in cognitive states, such as perceived workload, can predict future performance (Howard et al., 2021; Loft et al., 2018; Mracek et al., 2014). However, Griffiths et al. (2022) was the first study (to our knowledge) to have investigated the extent to which within-person variation in cognitive state predicts *RTM performance* (i.e. RTM accuracy and/or RTM RT to resolve aircraft conflicts missed by automation), an outcome variable of high relevance to adapting automation. Griffiths et al. found that increased trust in automation (relative to self; hereafter, rel. self) resulted in subsequently slower RTM RT (rel. self). Furthermore, those with higher trust (relative to sample; hereafter, rel. sample) had even slower RTM RT when their trust increased (rel. self) than those with lower trust (rel. sample). Between- and within-person fatigue interacted: for participants with low fatigue (rel. sample), increased fatigue (rel. self) quickened subsequent RTM RT, but not for those with average or high fatigue (rel. sample). Griffiths et al. found no effect of workload on RTM performance. While operator state predicted RTM RT with higher DOA, it remains untested whether operator state can predict subsequent RTM performance when individuals use lower DOA.

### Current Study

As reviewed, Griffiths et al. (2022) found that variation in some aspects of operator state predicted RTM RT. However, RTM performance deficits can also manifest in failure to intervene at all to a conflict missed by automation (RTM accuracy). For brevity below when making predictions, we refer to the two collectively as RTM performance.

Our first research question concerned whether operator state differentially predicts RTM performance as a function of DOA. If operator state differentially predicts RTM performance across DOA, then adaptive systems could be triggered by changes in operator state(s) as a function of the DOA being used. Our second research question concerned whether the findings in the higher DOA condition of the current study replicate Griffiths et al.’s (2022). It is critical to replicate (Pashler & Wagenmakers, 2012), particularly when resulting knowledge could be used to inform work design (Jones et al., 2010).

We measured between- and within-person variation in operator states and RTM performance when participants were provided higher or lower DOA in simulated ATC. Participants were responsible for conflict detection, which involved projecting future positions of aircraft to determine if any aircraft pairs would violate minimum separation (Loft et al., 2009). The higher DOA, as in Griffiths et al. (2022), automatically intervened to prevent conflicts. The lower DOA highlighted all aircraft travelling at the same altitude on converging flight paths, with participants responsible for changing aircraft altitude if they thought the pair would violate lateral separation. For both DOAs, the automation responded correctly to 24 conflicts (80% reliability), with participants required to manually intervene to six automation failures.

The task was paused every 3-min and participants responded to operator state questions (workload, fatigue, trust, task engagement). Automation failures occurred, on average, approximately 3-min after some question windows. At the within-person level, we used the average of ratings made to question windows 1-back and 2-back from each automation failure event (replicating Griffiths et al., 2022).

Our first hypothesis was that (H1) higher DOA would be associated with lower workload and poorer RTM performance than lower DOA (Onnasch et al., 2014). Additionally (H1) we predicted higher DOA may lower fatigue and task engagement (Bowden et al., 2024). Hierarchical linear modelling (HLM) was then used to examine between- and within-person effects of these operator states on RTM performance. First, we examined whether the effect of operator state on RTM performance differed across DOA. We then examined the effect of operator state on RTM performance for each DOA separately.

Griffiths et al. (2022) found that increased trust (rel. self) degraded RTM performance, but less so for those with low trust (rel. sample) compared to those with high trust (rel. sample). In retrospect, this finding was counterintuitive, perhaps indicating that increased trust was poorly calibrated (Carter et al., 2024; Lee & See, 2004). Nonetheless, we tentatively hypothesised the same interaction effect for between- and within-person trust for the current study’s higher DOA condition (H2). We then examined whether the interacting effects of higher trust (rel. sample) and increased trust (rel. self) exhibited the same pattern in the lower DOA condition (H3).

Griffiths et al. (2022) found that increased fatigue (rel. self) improved RTM performance for participants with low fatigue (rel. sample), but not for those with average or high fatigue. We hypothesised the same effects for higher DOA here (H4). Given we expected operators to be more fatigued with lower compared to higher DOA (H1), we examined whether the interacting effects of lower fatigue (rel. sample) and increased fatigue (rel. self) similarly degraded performance in the lower DOA condition (H5).

Griffiths et al. (2022) found no effect of workload on RTM performance. Thus, we expected that null finding for higher DOA here (H6). We expected that participants using lower DOA would report comparably higher workload (H1); subsequently, it is possible that increased workload (rel. self) and higher workload (rel. sample), or their interaction, may impact RTM performance under lower DOA conditions (H7).

Griffiths et al. (2022) did not measure task engagement, and to our knowledge, task engagement has not been examined as a predictor of RTM performance, but higher task engagement is theoretically linked to better performance (Cheyne et al., 2009). We expected lower task engagement with higher DOA (H1) and examined whether increased task engagement (rel. self) and higher task engagement (rel. sample), or their interaction, impacted RTM performance differentially across DOA (H8).

## Method

### Participants

The target sample size was based on the 102 participants that Griffiths et al. (2022) tested in their higher DOA condition. Thus, 204 undergraduate students from The University of Western Australia (UWA) participated in exchange for course credit or AUD$40 (121 female, *M*_age_ = 21.39; *SD*_age_ = 7.11, range = 17–63) and a performance-based incentive (AU$5–$20), with 102 assigned to the lower DOA and 102 to the higher DOA condition. This research complied with the American Psychological Association Code of Ethics and was approved by the UWA Human Research Ethics Office.

### ATC simulation

**Figure 1.**
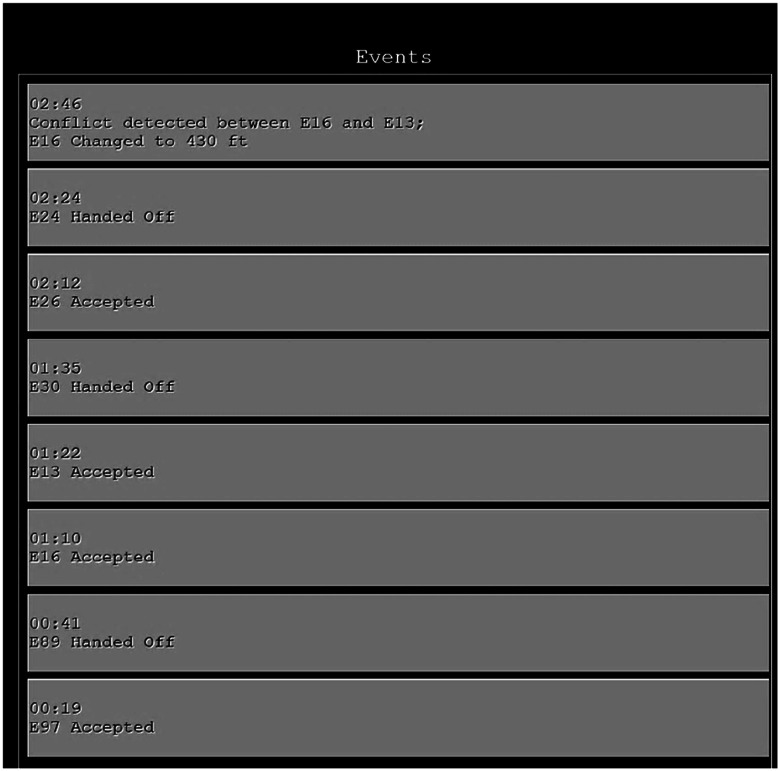
The event log contained actions performed by the participant or the automation and was updated as participants accepted and handed-off aircraft, intervened in aircraft conflicts, and when automation resolved conflicts. Log entries included the relevant aircraft, action, and time.

The ATC simulation (Fothergill et al., 2009) was presented on two 22-inch monitors and participants used a computer keyboard and mouse. The right-hand monitor contained flight strips and an event log (Figure 1). Flight strips contained aircraft callsign, altitude, and route. The event log displayed actions performed by the participant or the automation.

**Figure 2.**
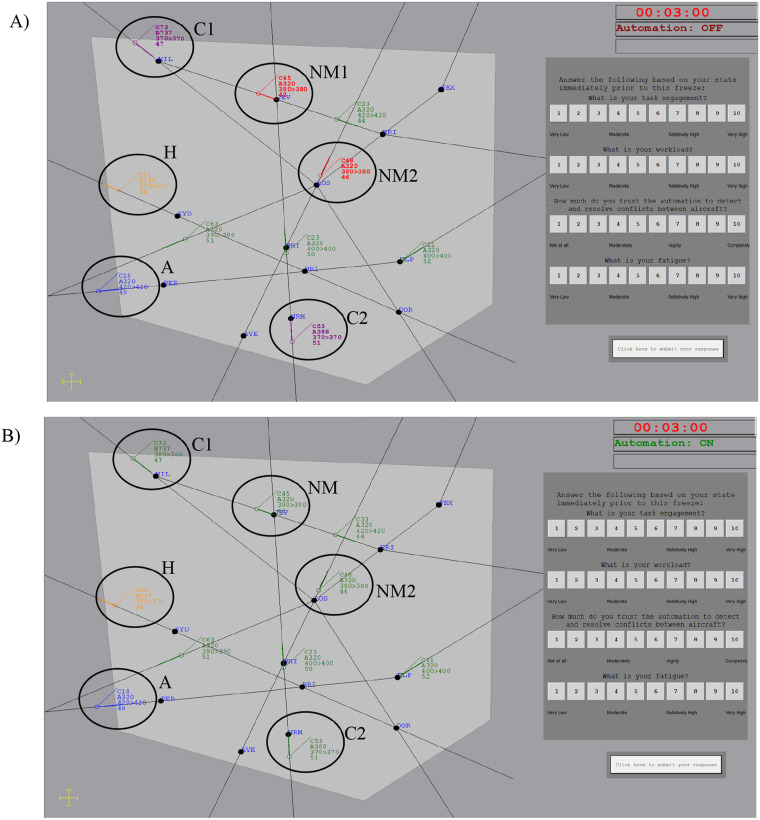
Map of the airspace (circles and labels added for clarity, not presented in-task). Aircraft can be seen requiring acceptance (A) and hand-off (H). The top right shows the scenario run-time (red when paused, black when running), and whether the conflict detection automation was active (this did not change when automation failed). The question window to the right of the screen contains four operator state questions. Panel a) Lower DOA highlighted all aircraft (alternating red and purple) flying on converging flight paths at the same flight level, which includes both conflicts (C1 and C2), and near-misses (NM1 and NM2). Panel b) Higher DOA automatically resolved conflicts by allocating one aircraft in the conflict pair to a new altitude (C1 has ascended to 38,000ft).

The left-hand monitor contained a sector map (Figure 2). The map displayed the inner controlled sector. Aircraft entered the controlled sector and travelled unidirectionally along flight paths denoted by black lines before exiting. Aircraft were denoted by icons with an attached projection line, indicating where the aircraft would be in 20s. An attached data block described each aircraft’s callsign (e.g. C53), aircraft type (e.g. A388), current and cleared altitude (e.g. 370 > 370 indicates cleared to fly at 37,000ft, flying at 37,000ft), and speed (e.g. 51 indicates 510 knots). Aircraft remained at the same speed and altitude unless instructed to ascend to avoid a conflict. The sector contained a median of eight aircraft at once.

Participants accepted and handed-off aircraft in and out of the sector. Aircraft flashed blue when 20s from the sector to request acceptance. Participants accepted aircraft by pressing the ‘A’ key and clicking on the aircraft. Accepted aircraft turned green when under participant control. When aircraft were 20s away from exiting the sector, they flashed orange to request hand-off. Participants handed-off aircraft by pressing the ‘H’ key and clicking on the aircraft. Handed-off aircraft turned black when no longer under participant control. Participants were notified of missed acceptances or hand-offs by an auditory alert.

Participants detected potential aircraft conflicts, defined as aircraft pairs that would violate minimum separation standards of 5Nm laterally and 1000ft vertically in the future. To detect conflicts, participants projected the future lateral separation of aircraft at the same altitude and on converging flightpaths. To intervene to prevent conflicts, participants clicked on both conflicting aircraft using a pop-up dialogue box. If the aircraft were in conflict, one aircraft ascended 1000ft and a notification was added to the event log. If selected aircraft were not in conflict, auditory false alarm alert sounded, and aircraft did not change altitude. If a conflict was not detected prior to minimum separation breach, an auditory alert sounded, and aircraft turned from green to yellow until separation was re-established. There were 30 conflicts (10 per scenario) and 18 ‘near-misses’ (6 per scenario). Near-miss aircraft pairs were at the same altitude and came close to (∼10s), but did not, violate lateral separation.

Participants were trained to resolve conflicts manually. Then in experimental scenarios, half of the participants were provided with lower DOA and the other half with higher DOA. In both conditions, automation failed to resolve (or highlight, in the case of lower DOA) six conflicts (two per scenario). Automation failures occurred on average 2-min 58s after some question windows (range: 2-min 10s – 3-min 47s). Participants were required to detect automation failures and intervene to prevent conflicts. Training instructions equally encouraged the accuracy and speed of conflict detection and intervention.

#### Lower degree of automation

Lower DOA (Figure 2(a)) highlighted all aircraft pairs travelling at the same flight level on converging paths in red or purple (alternating), which included both conflicts and near-misses. Participants were told that, as the automation highlighted all aircraft pairs at the same flight level on converging paths, being highlighted did not guarantee a conflict. Participants were required to assess aircraft future lateral separation to decide whether highlighted aircraft would conflict or not, and if the former, intervene to prevent conflicts. The lower DOA failed to highlight the same six conflicts as the higher DOA failed to resolve.

#### Higher degree of automation

The higher DOA (Figure 2(b)) was identical to Griffiths et al. (2022) and resolved conflicts upon the acceptance of the second aircraft in the pair. Automation resolved conflicts by instructing one of the aircraft to ascend 1,000ft. Participants were notified of this action in the event log. The higher DOA failed to resolve six conflicts.

### Operator state measures

**Figure 3.**
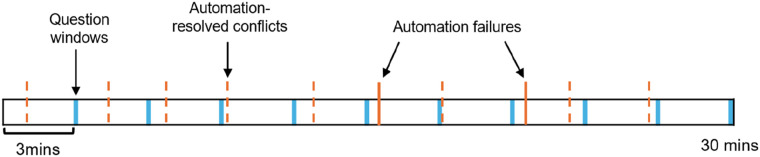
An example of question window and event timings presented to participants. Scenarios ran for 30 min, with question windows presented every 3 min. Of the 10 aircraft conflicts presented to participants, the automation failed to resolve two conflicts per scenario. Aircraft also needed to be accepted and handed-off throughout scenerious.

Participants responded to question windows every 3-min (10 times per scenario). A graphical representation of the timing of question windows relative to aircraft conflict events is presented in Figure 3. A 3-min interval was selected to ensure that operator state was measured prior to aircraft involved in a conflict first being displayed. Question windows did not prompt participants to upcoming automation failures, as only 20% of the windows preceded automation failure. Question window responses were self-paced (did not time-out).

Question windows appeared on the right-hand side of the sector while the task was paused (Figure 2). Aircraft remained visible during pauses to minimise task disruption (Chiappe et al., 2016). Response scales ranged from 1 to 10. Item presentation order (workload, fatigue, trust, task engagement) within the question window was counterbalanced across participants but consistent for each participant.

Participants completed multi-item measures of trust, fatigue, task engagement, and workload to provide convergent validity for the single-item measures. Trust in automation was measured post-task. Fatigue and task engagement were measured pre-task, as well as post-task, using the short version of the Dundee Stress State Questionnaire (DSSQ-SV; dimensions include fatigue, stress, and disengagement; Matthews et al., 2013). Workload was measured post-task using the NASA Task-load Index (NASA-TLX; Hart & Staveland, 1988). Table 1 presents the full list of operator state dependent variables.

**Table 1.** Descriptive Statistics for RTM Performance and Operator State Variables Measured In-Task and Post-Task, Split by DOA Condition.

Variables	Lower DOA			Higher DOA		
	Mean	SD	Range	Mean	SD	Range
Acceptance accuracy	1.00	0.01	0.94–1.00	1.00	0.003	0.99–1.00
Acceptance RT (s)	2.62	0.99	0.94–6.29	2.53	0.97	0.96–5.11
Hand-off accuracy	1.00	0.01	0.95–1.00	1.00	0.005	0.96–1.00
Hand-off RT (s)	2.89	1.04	1.02–6.36	2.72	1.03	0.94–5.55
Conflict false alarm rate	0.29	0.21	0.00–1.00	0.17	0.17	0.00–0.72
RTM accuracy	0.76	0.24	0.00–1.00	0.81	0.20	0.17–1.00
RTM RT (s)	90.5	27.3	16.7–168.9	102.6	25.5	29.3–157.4
In-task workload	4.39	1.76	1.00–9.07	3.92	1.99	1.03–9.03
In-task fatigue	5.23	2.11	1.00–10.00	5.08	2.38	1.00–10.00
In-task trust	4.39	1.78	1.17–8.50	6.16	1.96	1.43–9.53
In-task task engagement	5.84	2.07	1.03–10.00	5.23	2.25	1.00–10.00
Post-task trust	13.68	4.95	6.00–23.67	17.53	5.90	6.00–29.67
DSSQ: engagement	16.48	5.67	4.67–30.67	15.54	6.90	2.00–30.67
DSSQ: distress	9.87	4.73	0.00–20.67	8.98	4.61	0.00–22.00
DSSQ: worry	9.80	6.13	0.00–23.00	9.80	6.30	0.00–24.33
NASA-TLX	39.28	17.06	4.78–76.44	36.58	14.68	7.00–71.33

*Note.* RT (s) = response time in seconds; RTM = return to manual.

### Procedure

Participants first completed a separate 1-hr task examining manual conflict/non-conflict discrimination skill (data not reported here). This task sequentially presented 560 aircraft pairs travelling on converging flight paths for 3.5s each. Participants decided if each pair presented would conflict or not.

Participants then took a ∼10-min break in which they were randomly assigned to either the higher or lower DOA condition. They then completed a 25-min audio-visual training, 30-min manual ATC practice scenario, pre-DSSQ-SV questionnaire, 5-min training on condition-specific DOA, three 30-min experimental ATC scenarios with condition-specific DOA (∼5-min break between scenarios), and the trust inventory, DSSQ-SV, and NASA-TLX after each scenario.

### Data Analyses

Table 1 presents the full list of ATC performance metrics. Acceptance and hand-off accuracy was the proportion of aircraft successfully accepted/hand-off. Acceptance and hand-off RT was the time taken to accept/hand-off once an aircraft started flashing. Once accepted, participants could resolve conflicts manually in the practice scenario, or if they judged the automation had missed a conflict in experimental scenarios. RTM performance was measured by (a) the proportion of conflicts correctly resolved by the participant that automation failed to highlight or resolve (RTM accuracy) and (b) for those conflicts resolved, the time taken to resolve conflicts missed by automation (RTM RT). We also measured the conflict false alarm rate to near miss aircraft.

The data were nested such that each of the DOA conditions (between-person effect) contained 102 participants. Each participant produced six observations of RTM performance (within-person effect). HLM was used to account for the non-independence of observations (Raudenbush & Bryk, 2002), by including a random intercept term to account for multiple responses from each participant. Logistic HLM was run for the binary dependent variable of RTM accuracy (conflict resolved = 1, missed = 0), and HLM was run for RTM RT. Separate models were run for each operator state predictor of RTM performance, including centred between- and within-person effects and their interaction. Centred between-person effects reflected each participant’s average state across all 30 question window responses minus the sample mean for each state (e.g. Participant A’s mean workload – sample’s mean workload = Participant A’s centred between-person workload value). Within-person effects represented how each participant’s state varied from their own average state during the ATC scenarios (e.g., Participant A’s workload averaged over two questions windows prior [(T_5_ + T_6_)/2] – their mean workload across 30 question window responses = Participant A’s centred within-person workload value for proceeding automation failure).

We fit several HLMs. Model 1, the simpler model, included DOA, between-person operator state, within-person operator state, and the interaction of between- and within-person operator state. Model 2 was more complex by also testing for the moderating effect of DOA on the relationship between operator state and RTM performance, and so was identical to Model 1 with the addition of two- and three-way interaction terms between DOA, between-, and within-person operator states. Model 1 and 2 equations can be found in the appendices.

Next, a model was fit for operator states predicting RTM performance for each DOA condition separately, to replicate the Griffiths et al. (2022) higher DOA analysis. All analyses were conducted in R/lme4 package (Bates et al., 2015; R Core Team, 2021).

## Results

Table 1 presents descriptive statistics as a function of DOA. Aircraft acceptance and hand-off accuracy were at ceiling, and with no differences between DOA conditions for acceptance RTs, *t* < 1, or hand-off RTs, *t*(202) = 1.15, *p* = .25. Participants made more conflict false alarms in the lower DOA condition, *t*(190.51) = 4.47, *p* < .001, *d* = .63.

There was no difference in RTM accuracy between the higher and lower DOA conditions, *t*(195.85) = 1.49, *p* = .14. However, consistent with H1, participants had slower RTM RT in the higher DOA condition, *t*(200) = 3.27, *p* < .01, *d* = .46.

Trust was higher in the higher DOA condition, *t*(202) = 6.73, *p* < .001, *d* = .95. Both workload, *t*(202) = 1.79, *p* = .08, and task engagement, *t*(202) = 2.00, *p* = .05, trended toward being higher in the lower DOA condition (partially consistent with H1). Inconsistent with H1, fatigue did not differ between DOA conditions, *t* < 1.

Table 2 presents between-person correlations. Moderate to strong correlations (using Cohen’s [1992], correlation effect size guidelines) between post-task questionnaires and in-task measures in Table 2 demonstrated convergent validity for the in-task workload, engagement, and trust measures.

**Table 2.** Between-Person Correlation Matrix (Data Collapsed Across DOA).

Variable	1.	2.	3.	4.	5.	6.	7.	8.	9.	10.	11.	12.	13.	14.	15.
1. Acceptance accuracy															
2. Acceptance RT (s)	**−0.27**														
3. Hand-off accuracy	**0.56**	**−0.38**													
4. Hand-off RT (s)	**−0.30**	**0.90**	**−0.41**												
5. Conflict false alarm rate	−0.08	0.03	−0.09	0.03											
6. RTM accuracy	0.14	**−0.34**	**0.26**	**−0.35**	−0.12										
7. RTM RT (s)	0.06	**0.31**	0.005	**0.21**	**−0.34**	**−0.26**									
8. In-task workload	−0.06	0.07	−0.01	0.03	**0.14**	**−0.14**	0.01								
9. In-task fatigue	**−0.18**	**0.26**	−0.11	**0.23**	0.10	**−0.19**	0.08	**0.34**							
10. In-task trust	−0.02	0.003	−0.09	−0.07	−0.02	0.01	0.06	−0.09	−0.07						
11. In-task engagement	0.03	**−0.21**	0.11	**−0.20**	0.08	0.09	**−0.14**	**0.38**	**−0.31**	−0.04					
12. Post-task trust	−0.05	0.11	−0.12	0.03	0.02	−0.05	0.11	**−0.16**	0.01	**0.79**	**−0.15**				
13. DSSQ: engagement	0.13	**−0.24**	0.10	**−0.24**	0.04	0.06	−0.12	0.10	**−0.58**	−0.07	**0.68**	−0.08			
14. DSSQ: distress	**−0.18**	**0.18**	−0.13	0.12	**0.19**	**−0.19**	0.07	**0.33**	**0.37**	−0.10	**−0.18**	−0.02	**−0.33**		
15. DSSQ: worry	−0.02	0.08	−0.07	0.08	0.03	−0.12	0.07	−0.06	0.07	−0.05	**−0.16**	0.08	**−0.14**	**0.31**	
16. NASA-TLX	0.01	−0.03	0.02	−0.02	**0.18**	**−0.17**	0.03	**0.55**	**0.15**	**−0.16**	**0.24**	**−0.22**	**0.14**	**0.41**	0.05

*Note.* RTM = Return to Manual, RT (s) = Response time in seconds. Significant values are bolded (two-tailed). Weak, moderate, and strong correlation effect sizes are 0.10, 0.30, and 0.50, respectively (Cohen, 1992).

### Hierarchical linear modelling

Likelihood ratio tests were used to compare Models 1 and 2. When model comparisons reveal comparable models in terms of fit, the more parsimonious model is selected (Whittaker & Furlow, 2009). The simpler Model 1 fit the data comparably to the more complex Model 2 that additionally tested for the moderating effect of DOA on the relationship between operator state and RTM performance. Thus, we found no evidence that accounting for the effect of operator state as a function of DOA improved the prediction of RTM performance (no support for H3, H5, H7, H8). Therefore Model 1 (the simpler model) is reported below. The random effect parameters of Model 1 (not reported in text below), Model 2, and model comparison outcomes are presented in the appendices.

Model 1 results are presented in Table 3. Intra-class correlations indicated substantial within-person variation in RTM performance: 63.03% for RTM accuracy and 84.72% for RTM RT. This within-person variation in RTM performance could potentially be predicted by variation in operator state prior to automation failures. The main effects of DOA on operator state variables and RTM performance have been addressed above and are thus not discussed in-text for the models reported below.

**Table 3.** Results of Hierarchical Linear Model 1: Between- and Within-Person Effects of Workload, Task Fatigue, Trust in Automation, and Task Engagement on RTM Accuracy and RT.

Operator State	Predictor	RTM Accuracy Model	RTM RT Model
WORKLOAD	Intercept	**1.51 (.16)**	**87.83 (2.63)**
	Between	−.10 (.06)	.20 (.97)
	Within	**−.56 (.11)**	3.63 (1.95)
	DOA	.30 (.21)	**13.05 (3.63)**
	Between × within	.06 (.04)	−1.19 (.77)
	*X*^2^ - M1 vs. null	**33.71**	**18.79**
FATIGUE	Intercept	**1.49 (.16)**	**88.70 (2.66)**
	Between	**−.12 (.05)**	.65 (.81)
	Within	**−.34 (.09)**	.40 (1.53)
	DOA	.29 (.20)	**13.40 (3.61)**
	Between × within	.06 (.06)	.10 (.59)
	*X*^2^ - M1 vs. null	**21.77**	**14.02**
TRUST	Intercept	**1.48 (.17)**	**87.84 (2.77)**
	Between	−.05 (.06)	−.17 (.96)
	Within	**.31 (.09)**	−.28 (1.59)
	DOA	.46 (.24)	**13.51 (4.01)**
	Between × within	.02 (.03)	−.49 (.50)
	*X*^2^ - M1 vs. null	**43.16**	**14.46**
ENGAGEMENT	Intercept	**1.37 (.15)**	**89.43 (2.66)**
	Between	.07 (.05)	−1.28 (.85)
	Within	−.06 (.10)	−2.64 (1.72)
	DOA	.35 (.20)	**12.64 (3.62)**
	Between × within	.005 (.04)	.41 (.67)
	*X*^2^ - M1 vs. null	6.94	**17.03**

*Note.* Values represent the unstandardised regression estimates, and parenthetical values indicate standard errors. Significant values are bolded (two-tailed).

Contrary to Griffiths et al. (2022), there were no significant interactions between within- and between-person operator state variables in predicting RTM performance, and thus only the main effects of operator state on RTM performance are reported below. The regression coefficients (betas) in Tables 3 and 4 represent unstandardised effect size measures, and we use these to calculate and comment on practical effect sizes.

**Table 4.** Results of Hierarchical Linear Models Examining Between- and Within-Person Effects of Workload, Task Fatigue, Trust in Automation, and Task Engagement on RTM Accuracy and RTM RT (for Higher and Lower DOA) Separately, and Comparison to the Griffiths et al. (2022) Higher DOA Condition.

	Lower DOA		Higher DOA		Higher DOA (from Griffiths et al., 2022)	
	RTM accuracy	RTM RT (s)	RTM accuracy	RTM RT (s)	RTM accuracy	RTM RT (s)
Workload						
Intercept	**1.63** (**.19)**	**87.56** (**2.81)**	**1.73** (**1.70)**	**100.64** (**2.57)**	**2.06 (.21)**	**105.64 (2.69)**
Between	**−.19** (**.09)**	1.66 (1.55)	−.02 (.07)	−.92 (1.22)	−.05 (.12)	−2.97 (2.46)
Within	**−.70** (**.17)**	**5.76** (**2.90)**	**−.47** (**.14)**	1.82 (2.67)	−.15 (.16)	.54 (1.84)
Between × within	.11 (.06)	−1.41 (1.23)	.02 (.06)	−1.13 (1.08)	.04 (.05)	1.08 (.85)
Fatigue						
Intercept	**1.57** (**.19)**	**88.97** (**2.87)**	**1.72** (**.16)**	**101.71** (**2.59)**	**1.97 (.21)**	**107.95 (2.61)**
Between	**−.16** (**.07)**	0.69 (1.30)	−.09 (.06)	.54 (1.03)	−.12 (.10)	.00 (1.41)
Within	**−.54** (**.13)**	1.03 (2.29)	−.16 (.11)	−.39 (2.10)	−.16 (.12)	−1.72 (1.91)
Between × within	.06 (.05)	.26 (.83)	.07 (.04)	−.21 (.87)	−.01 (.03)	**1.51 (.51)**
Trust						
Intercept	**1.55** (**.21)**	**87.12** (**3.15)**	**1.82** (**.19)**	**101.50** (**2.83)**	**2.10 (.29)**	**107.90 (2.68)**
Between	−.10 (.09)	−.47 (1.49)	−.02 (.07)	.06 (1.24)	.17 (.13)	1.58 (1.80)
Within	**.43** (**.13)**	−1.49 (2.36)	.15 (.12)	.47 (2.23)	.23 (.15)	**6.47 (2.21)**
Between × within	.01 (.04)	−.92 (.79)	.06 (.03)	−.27 (.69)	.01 (.04)	**1.67 (.59)**
Engagement						
Intercept	**1.40** (**.17)**	**88.81** (**2.90)**	**1.68** (**.16)**	**102.08** (**2.54)**	NA	NA
Between	.05 (.07)	.04 (1.33)	.08 (.06)	**−2.36** (**1.08)**	NA	NA
Within	.10 (.14)	−1.52 (2.54)	−.24 (.15)	−3.39 (2.39)	NA	NA
Between × within	.004 (.05)	.13 (.95)	−.02 (.06)	.73 (.98)	NA	NA

*Note.* Values represent the unstandardised regression estimates, and parenthetical values indicate standard errors. Significant values are bolded (two-tailed). NA = non-applicable.

#### Workload

Between-person workload did not predict RTM accuracy. At the within-person level, workload predicted RTM accuracy such that participants were 1.75 times less likely (rel. self) to resolve conflicts missed by automation for each unit increase in workload (rel. self). Neither between- nor within-person workload predicted RTM RT.

#### Fatigue

At the between-person level, for each unit higher-than-average a participant’s fatigue (rel. sample), they were 1.13 times less likely to resolve conflicts missed by automation (rel. sample). At the within-person level, fatigue predicted RTM accuracy such that participants were 1.40 times less likely (rel. self) to resolve conflicts missed by automation for each unit increase in fatigue (rel. self). Neither between- nor within-person fatigue predicted RTM RT.

#### Trust in automation

Between-person trust did not predict RTM accuracy. At the within-person level, trust (rel. self) predicted RTM accuracy such that participants were 1.36 times *more* likely (rel. self) to resolve conflicts missed by automation for each unit increase in trust (rel. self). Neither between- nor within-person trust in automation predicted RTM RT.

#### Task engagement

Neither between- nor within-person task engagement predicted RTM performance.

#### Predictive Validity of Operator States for Each DOA

Table 4 presents the predictive validity of operator states on RTM accuracy and RTM RT, for lower and higher DOA conditions separately. For the higher DOA model, we also include the HLM outcomes for Griffiths et al. (2022) to compare their unstandardised effect sizes from their equivalent condition to the higher DOA condition of the current study.

For the higher DOA model, the findings of Griffiths et al. (2022) of the predictive relationships between trust (H2) and fatigue (H4) on RTM performance were not replicated. Also, contrary to the Griffiths et al. null finding and thus H6, workload predicted RTM accuracy such that participants were 1.51 times less likely (rel. self) to resolve conflicts missed by automation for each unit increase in workload (rel. self). Although not examined by Griffiths et al., for each unit higher-than-average a participant’s task engagement (rel. sample), participants were 3.39s faster to resolve conflicts missed by automation (rel. sample).

For the lower DOA model, for each unit higher-than-average a participant’s workload (rel. sample), they were 1.21 times less likely (rel. sample) to resolve conflicts missed by automation. At the within-person level, workload predicted RTM performance such that participants were 2.02 times less likely to resolve conflicts missed by automation, and 5.76s slower (rel. self) to do so for each unit increase in workload (rel. self). Participants were 1.71 times less likely to resolve conflicts missed by automation (rel. self) for every unit increase in fatigue (rel. self). At the between-person level, for each unit higher-than-average a participant’s fatigue (rel. sample), they were 1.17 times less likely to resolve conflicts missed by automation (rel. sample). Trust predicted RTM accuracy such that participants were 1.54 times *more* likely (rel. self) to resolve conflicts missed by automation for each unit increase in a participant’s trust (rel. self).

## Discussion

Adaptive work systems are a potential solution to balance the benefits/costs of using different DOAs (Feigh et al., 2012; Kaber & Riley, 1999); for example, by proactively shifting DOA function allocation or making other adaptations (e.g. task scheduling) based on cognitive states of operators. Griffiths et al. (2022) presented initial evidence that variation in operator state can predict RTM performance when higher DOA fails, namely, the time taken to resolve conflicts missed by automation (RTM RT). We aimed to: (1) examine whether variation in operator state differentially predicted RTM performance (accuracy and/or RT) as a function of DOA and (2) determine if Griffiths et al.’s higher DOA outcomes replicated.

The Onnasch et al. (2014) meta-analysis indicated that as DOA increases, benefits to workload and routine performance increase, but at potential cost to SA/RTM performance. Partially consistent with Onnasch et al., participants in the higher DOA condition were 12.1s slower to resolve conflicts missed by automation than participants in the lower DOA condition, but there was only a (non-significant) trend toward lower workload with higher DOA. We did not measure SA or routine conflict detection performance (although more conflict detection false alarms were made by the lower DOA condition).

Consistent with H1, and as mentioned above, participants in the higher DOA condition had poorer RTM performance. Partially consistent with H1, there was a (non-significant) trend toward lower workload and lower task engagement with higher DOA.

Contrary to H1, fatigue did not significantly differ across DOA. Participants reported higher trust when using higher DOA. Given these differences in outcome variables between DOA conditions, we speculated that changes in operator state could differentially impact RTM performance as a function of DOA. We found no support for this contention. Additionally accounting for interactions between DOA and operator state did not improve the prediction of RTM performance, and the simpler model that included DOA, between-person operator state, within-person operator state, and the interaction between- and within-person operator state, was equally predictive. This simpler model indicated that increased within-person workload and fatigue, and higher between-person fatigue, decreased the probability that participants resolved conflicts missed by automation. Increased within-person trust in the automation on the other hand was *beneficial* to subsequent RTM accuracy. We now discuss the impact of operator state on RTM performance separately for each DOA condition to allow a direct comparison with the Griffiths et al. (2022) higher DOA condition.

### Higher Degree Automation

With higher DOA, Griffiths et al. (2022) found that increased fatigue (rel. self) benefitted RTM RT (5.24s faster for each within-person unit increase in fatigue), but only for those with low fatigue (rel. sample). We did not replicate this; fatigue had no impact on RTM performance. Griffiths et al. also found increased trust (rel. self) was detrimental to subsequent RTM RT, particularly for participants with high trust (rel. sample) (9.79s slower with each within-person unit increase in trust). This was not replicated as we found no impact of trust on RTM performance. We did find that with higher DOA, higher task engagement (rel. sample) improved RTM RT (rel. sample). This effect is consistent with more engaged participants being less complacent (Neubauer et al., 2014; Saxby et al., 2013). We also found with higher DOA, increased workload (rel. sample) degraded RTM accuracy (rel. sample). Griffiths et al. did not find this, and our finding contrasts with the premise in the literature that higher DOA can degrade RTM performance because it causes operator underload, potentially shrinking attentional resources (Young & Stanton, 2002a; 2002b) or otherwise decreasing monitoring of automation (Parasuraman & Manzey, 2010; Wickens et al., 2015).

While this lack of replication was unexpected, it is crucial that such findings are published to avoid the potential ‘file drawer’ problem, such that cumulative knowledge can be used to generate precise meta-analytic effect size estimates for evidence-based work design interventions (Cumming, 2012; Jones et al., 2010). Nonetheless, when replication fails and other results differ across studies, it is critical to consider points of methodological difference. The two studies used the same undergraduate student samples of comparable average age and gender. Unlike Griffiths et al. (2022), the current study included a 1-hr manual conflict detection task before the main experiment. However, based on the comparative raw data presented in Table 5, we do not believe this additional task accounts for the lack of replication, with RTM accuracy and RT equivalent across the two studies. Current study participants reported higher in-task fatigue than in Griffiths et al., and given it was only participants with low fatigue (rel. sample) whose RTM performance was positively impacted by increased fatigue (rel. self) in Griffiths et al., this could have contributed to the lack of fatigue effects on RTM here. Current study participants also reported higher in-task workload, which may have increased the current study’s ability to detect a negative impact of increased workload (rel. self) on RTM accuracy.

**Table 5.** Comparison of Common Raw Data Across Griffiths et al. (2022) and the Current Study (Higher DOA Condition).

Variable	Griffiths et al. (2022)	Current Study (Higher DOA)	Independent Groups *t*-test
	Mean (SD)	Mean (SD)	
Acceptance accuracy	1.00 (.01)	1.00 (.01)	*t* < 1
Acceptance RT (s)	2.29 (.84)	2.53 (.97)	*t*(202) = 1.89, *p* = .06
Hand-off accuracy	1.00 (.01)	1.00 (.005)	*t* < 1
Hand-off RT (s)	2.45 (.91)	2.72 (1.03)	*t*(202) = 1.98, *p* = .05
Conflict false alarm rate	.15 (.18)	.17 (.17)	*t* < 1
RTM accuracy	.83 (.21)	.81 (.20)	*t* < 1
RTM RT (s)	104.23 (44.90)	102.6 (25.5)	*t* < 1
**In-task workload**	**3.30** (**1.37)**	**3.92** (**1.99)**	***t*(202) = 2.59, *p* = .01**
**In-task fatigue**	**4.23** (**1.70)**	**5.08** (**2.38)**	***t*(202) = 2.94, *p* = .004**
In-task trust	6.26 (1.37)	6.16 (1.96)	*t* < 1
Post-task trust	16.57 (5.01)	17.53 (5.90)	*t*(202) = 1.25, *p* = .21
**DSSQ: engagement**	**18.20** (**5.77)**	**15.54** (**6.90)**	***t*(202) = 2.99, *p* = .003**
DSSQ: distress	9.96 (4.55)	8.98 (4.61)	*t*(202) = 1.53, *p* = .13
DSSQ: worry	10.38 (5.88)	9.80 (6.30)	*t* < 1
NASA-TLX	39.88 (18.20)	36.58 (14.68)	*t*(202) = 1.43, *p* = .16

*Note.* Both the Griffiths et al. (2022) and Current Study (Higher DOA) samples had *N* = 102. s = seconds.

### Lower Degree Automation

With lower DOA, higher workload (rel. sample) degraded RTM accuracy (rel. sample) and increased workload (rel. self) degraded RTM RT (rel. self). Additionally, fatigue degraded RTM accuracy when a participant’s fatigue was higher than the sample average, or if fatigue increased above their own average. While average fatigue did not differ between DOA conditions, workload was moderately positively correlated with fatigue at both between- and within-person levels for both DOA conditions, as well as in Griffiths et al. (higher DOA only, 2022). Collectively, these outcomes suggest that operators are potentially more likely to suffer from RTM performance decrements when they feel more overloaded compared to others, or when they are using more cognitive capacity than they typically do.

Increased within-person trust (i.e., rel. self) predicted improved RTM accuracy in the lower DOA condition. The Griffiths et al. (2022) finding that increased trust (rel. self) degraded RTM performance (but note this was under higher DOA conditions), in retrospect, was counterintuitive. The current finding of improved RTM accuracy with increased trust when using lower DOA is more in line with conceptualisations that increased trust reflects operators’ confidence in their ability to predict automation performance (Carter et al., 2024; Lee & See, 2004). If increased trust indeed reflected more calibrated automation error prediction (i.e., prediction of whether lower DOA would highlight the correct aircraft pairs), it is logical that increased trust led to better RTM performance.

### Limitations, Future Research, and Conclusions

Operator state did not differentially impact RTM performance as a function of DOA. We do not believe this is the result of a weak DOA manipulation; the lower DOA aided information processing (stage 1/2: highlighting, integration) and the higher DOA implemented actions (stage 4; see Onnasch et al., 2014 for the ‘critical boundary’ across which Lumberjack Model predictions are predicted to hold true; but also see related criticisms by Jamieson & Skraaning, 2020). In line with this, we at least partially replicated some of the effects expected with increased DOA (better RTM performance and a trend towards higher workload with lower DOA).

Many of our higher DOA findings regarding operator state and RTM performance did not replicate Griffiths et al. (2022), highlighting the complexity associated with predicting RTM performance from perceived cognitive states. Further research is required to determine the direction/consistency in which operator states impact RTM performance, including potential boundary conditions, and in high-fidelity settings and with experts (Jamieson & Skraaning, 2020). We acknowledge that the RTM transition we used simplifies real work conditions, where operators may not fully RTM if automation fails. Future studies should examine whether operator state predicts performance with other automation transitions (e.g. incremental, instead of full, and automated support reduction).

We used self-report measures of cognitive state, which may not be feasible in operational settings. Although there are concerns about whether passively worn physiological measures are reliable and valid indicators of cognitive state (Charles & Nixon, 2019), future research should continue exploring real-time operator state measures, including eye-tracking (Lu & Sarter, 2019) and heart-rate variability (Michailovs et al., 2024). One notable measure of cognitive state not included here is SA. While SA measurement was beyond scope due to experimental duration/task interruption constraints, measuring SA would be valuable given its centrality to the Lumberjack Model. The current findings that operator states can predict RTM performance provide another step toward understanding how automation can be triggered to proactively adapt. Collapsed across DOA, increased fatigue (rel. sample and rel. self) and increased workload (rel. self) decreased the probability that participants resolved conflicts missed by automation. Increased trust in the automation (rel. self) on the other hand was beneficial to subsequent RTM accuracy.

## Key Points

• Investigated whether variability in operator state (workload, fatigue, trust, task engagement) predicted return-to-manual (RTM) performance after higher- or lower-degree automation failed to resolve aircraft conflicts in simulated air traffic control.
• We replicated previous findings that RTM performance was better when using lower compared to higher degree of automation (DOA). Operator states did not differentially predict RTM performance in the higher compared to the lower DOA conditions.
• Collapsed across DOA, an increase in a participants self-reported workload or fatigue, or a decrease in trust in automation, was detrimental to their subsequent RTM performance. RTM performance was poorer for participants with higher average fatigue compared to others. Task engagement did not predict RTM performance.
• Variability in operator state is a potential proactive method to adapt work systems in line with an operator’s projected future performance.

## Note

1. The majority of the studies included in the Onnasch et al. (2014) meta-analysis did not refer to “DOA” (many were published before the concept of DOA was introduced by Wickens et al., 2010). For each study, Onnasch et al. (2014) defined DOA as an increasing rank reflecting increased DOA via either stages or levels.

## Appendix A Full Hierarchical Linear Model Results – RTM Accuracy

Null model: y ∼ 1 + (1 | Participant)

Model 1 equation: y ∼ 1 + Within-person effect x Between-person effect + DOA + (1 | Participant)

Model 2 equation: y ∼ 1 + Within-person effect x Between-person effect x DOA + (1 | Participant)

**Table:**

Model	Fixed Effects Unstandardised Estimates (*se*)								Random Effect Variance (*sd*)	Likelihood Ratio Test
	Intercept	Between	Within	DOA	Between × within	Between × DOA	Within × DOA	B × W × DOA	Participant	*X* ^2^
Null	1.53 (.11)***								.86 (.95)	
Workload										
1	1.51 (.16)***	−.10 (.06)	−.56 (.11)***	.30 (.21)	.06 (.04)				.97 (.98)	33.71 ***
2	1.57 (.17)***	−.19 (.08)*	−.67 (.16)***	.22 (.23)	.10 (06)	.16 (.11)	.19 (.21)	−.08 (.09)	.91 (.96)	2.78
Fatigue										
1	1.49 (.16)***	−.12 (.05)**	−.34 (.09)***	.29 (.20)	.06 (.06)				.88 (.94)	21.77 ***
2	1.51 (.16)***	−.15 (.07)*	−.51 (.13)***	.28 (.22)	.05 (.05)	.06 (.09)	.34 (.17)*	.03 (.06)	.88 (.94)	4.49
Trust										
1	1.48 (.17)***	−.05 (.06)	.31 (.09)***	.46 (.24)	.02 (.03)				1.02 (1.01)	43.16***
2	1.48 (.18)***	−.10 (.08)	.41 (.13)**	.42 (.25)	.01 (.04)	.08 (.17)	−.25 (.18)	.06 (.05)	1.04 (1.02)	5.51
Engagement										
1	1.37 (.15)***	.07 (.05)	−.06 (.10)	.35 (.20)	.005 (.04)				.86 (.93)	6.94
2	1.36 (.16)***	.05 (.07)	.10 (.14)	.38 (.22)	−.002 (.05)	.02 (.09)	−.35 (.20)	−.02 (.08)	.86 (.93)	4.67

*Note. p* < .05*, *p* < .01**, *p* < .001***; (two-tailed) likelihood ratio test compares each model to the simpler model.

## Appendix B Full Hierarchical Linear Model Results – RTM RT

Null model: y ∼ 1 + (1 | Participant)

Model 1 equation: y ∼ 1 + Within-person effect x Between-person effect + DOA + (1 | Participant)

Model 2 equation: y ∼ 1 + Within-person effect x Between-person effect x DOA + (1 | Participant)

**Table:**

Model	Fixed effects unstandardised estimates (se)								Random effect variance (*sd*)	Likeli-hood ratio test
	Intercept	Between	Within	DOA	Between × within	Between × DOA	Within × DOA	B × W × DOA	Participant	*X* ^2^
Null	95.45 (1.86)***								316.50 (17.79)	
Workload										
1	87.83 (2.63)***	.20 (.97)	3.63 (1.95)	13.05 (3.63)***	−1.19 (.77)				274.30 (16.56)	18.79***
2	87.51 (2.68)***	1.66 (1.47)	5.76 (2.82)*	13.19 (3.80)***	−1.37 (1.10)	−2.58 (1.96)	−3.94 (3.93)	.26 (1.56)	272.20 (16.50)	2.01
Fatigue										
1	88.70 (2.66)***	.65 (.81)	.40 (1.53)	13.40 (3.61)***	.10 (.59)				273.00 (16.52)	14.02**
2	88.93 (2.74)***	.69 (1.24)	1.01 (2.22)	12.84 (3.86)**	.29 (.81)	−.12 (1.65)	−1.35 (3.10)	−.47 (1.20)	274.90 (16.58)	.41
Trust										
1	87.84 (2.77)***	−.17 (.96)	−.28 (1.59)	13.51 (4.01)***	−.49 (.50)				273.30 (16.53)	14.46**
2	87.01 (3.02)***	−.47 (1.42)	−1.47 (2.29)	14.52 (4.23)***	−.95 (.77)	.51 (1.93)	1.91 (3.25)	.66 (1.04)	272.80 (16.52)	.99
Engagement										
1	89.43 (2.66)***	−1.28 (.85)	−2.64 (1.72)	12.64 (3.62)***	.41 (.67)				266.60 (16.33)	17.03***
2	88.71 (2.75)***	.04 (1.25)	−1.54 (2.46)	13.42 (3.85)***	.13 (.92)	−2.41 (1.70)	−1.79 (3.49)	.61 (1.36)	265.20 (16.29)	2.20

*Note. p* < .05*, *p* < .01**, *p* < .001***; (two-tailed) likelihood ratio test compares each model to the simpler model.
